# Comparative analysis of complete chloroplast genome sequences of two tropical trees *Machilus yunnanensis* and *Machilus balansae* in the family Lauraceae

**DOI:** 10.3389/fpls.2015.00662

**Published:** 2015-08-25

**Authors:** Yu Song, Wenpan Dong, Bing Liu, Chao Xu, Xin Yao, Jie Gao, Richard T. Corlett

**Affiliations:** ^1^Center for Integrative Conservation, Xishuangbanna Tropical Botanical Garden, Chinese Academy of SciencesMengla, China; ^2^Graduate School of the Chinese Academy of SciencesBeijing, China; ^3^Peking-Tsinghua Center for Life Sciences, Academy for Advanced Interdisciplinary Studies, Peking UniversityBeijing, China; ^4^State Key Laboratory of Systematic and Evolutionary Botany, Institute of Botany, Chinese Academy of SciencesBeijing, China; ^5^Key Laboratory of Tropical Forest Ecology, Xishuangbanna Tropical Botanical Garden, Chinese Academy of SciencesMengla, China

**Keywords:** mutation, genome, chloroplast, *Machilus*, Lauraceae

## Abstract

*Machilus* is a large (c. 100 sp.) genus of trees in the family Lauraceae, distributed in tropical and subtropical East Asia. Both molecular species identification and phylogenetic studies of this morphologically uniform genus have been constrained by insufficient variable sites among frequently used biomarkers. To better understand the mutation patterns in the chloroplast genome of *Machilus*, the complete plastomes of two species were sequenced. The plastomes of *Machilus yunnanensis* and *M. balansae* were 152, 622 and 152, 721 bp, respectively. Seven highly variable regions between the two *Machilus* species were identified and 297 mutation events, including one micro-inversion in the *ccsA-ndhD* region, 65 indels, and 231 substitutions, were accurately located. Thirty-six microsatellite sites were found for use in species identification and 95 single-nucleotide changes were identified in gene coding regions.

## Introduction

The genus *Machilus* in the family Lauraceae includes nearly 100 tree species distributed in tropical and subtropical East and South Asia, with most species in China (Tang et al., 2010). *Machilus* species are known for their high-quality wood. Although the relationships among the genera traditionally recognized within the monophyletic ‘*Persea* group’ are still unclear, *Machilus* (with two misplaced species of *Phoebe*) forms a distinct, monophyletic, Asian clade (Chen et al., 2009; Rohwer et al., 2009; Li et al., 2011). At the species level, however, the reported nuclear *ITS* and *LEAFY* intron II sequences failed to resolve the phylogenetic and species identification problems in this genus. Rohwer et al. (2009) suggest that the extremely low genetic variation among species of *Machilus* could be explained by recent species differentiation and/or a greatly decreased substitution rate within the genus. Besides nuclear sequences, 14 chloroplast genomic markers (*matK, trnK, accD, ndhJ, psbC-trnS, rpoB, rpoC1, trnD-trnT2, trnH-trnK, psbA-trnH, psbB-psbH, trnS-trnG, rpoB-trnC*, and *trnS-trnfM*) also failed to resolve either the phylogenetic problems within the *Persea* group or species delineation within *Machilus* (Rohwer, 2000; Rohwer and Rudolph, 2005; Rohwer et al., 2009; Li et al., 2011). All of these results showed very little variation in those chloroplast genomic markers. This raises the question, are there any useful sequences for the phylogenetic classification of *Machilus* species in the chloroplast genome?

The chloroplast genome is more conserved than the nuclear genome in plants, but many mutation events in the chloroplast DNA sequence have been identified, including indels, substitutions, and inversions (Ingvarsson et al., 2003). At a high taxonomic level, a 22 kb DNA inversion event was used to confirm that the Barnadesioideae is the most basal lineage in the Asteraceae (Jansen and Palmer, 1987), and three DNA inversion events composed a nested set of phylogenetic characters to clarify the close relationship between the Poaceae and Joinvilleaceae (Doyle et al., 1992). At a low taxonomic level in ginseng, the DNA polymorphism rates of indels and SNPs between *Panax ginseng* and *P. notoginseng* were 0.40% (Dong et al., 2014), and 0.20% among four chloroplast genomes of different Chinese ginseng strains (Zhao et al., 2015). In rice, the DNA polymorphism rate of indels and SNPs between *Oryza sativa* and *O. nivara* were 0.02% (Masood et al., 2004), and 0.07% between *O. sativa indica* and *O. sativa japonica* (Tang et al., 2004). All of these results show that variable characters exist among the chloroplast genomes at the species level.

Here, two species of *Machilus* (Lauraceae) were selected to determine the entire chloroplast genome sequences. *Machilus yunnanensis* Lecomte is distributed at high altitudes in Yunnan, Sichuan, and Tibet of SW China (Wei and Werff, 2008), while *M. balansae* (Airy Shaw) F. N. Wei and S. C. Tang occurs mainly at low elevations in North Vietnam (Tang et al., 2010). By comparing these two complete chloroplast genomes we will try to answer the following questions: (1) What is the size range of chloroplast genomes in *Machilus*? (2) Which types of mutation events occurred in chloroplast genomes of *Machilus*? (3) Is there any highly variable region in the chloroplast genomes of *Machilus*? Comparisons were also made with the recently published chloroplast genome of *Cinnamomum kanehirae* (Wu et al., 2015).

## Materials and Methods

### DNA Extraction and Sequencing

We collected young leaves of *M. yunnanensis* and *M. balansae* from single seedlings growing in the nursery of the Xishuangbanna Tropical Botanical Garden (XTBG) on May 20, 2014. We also collected fruiting branches of both mother trees (Supplementary Figure S1) and compared them with the types to confirm their identifications (Supplementary Figure S2). Genomic DNA was extracted from 1 g fresh leaves using the mCTAB method (Li et al., 2013). Both genomes were sequenced following Dong et al. (2013), and their 138 pair specific primers were used to bridge gaps in the plastomes.

### Chloroplast Genome Assembling and Annotation

Sanger sequence reads were proofread and assembled with Sequencher 4.10 (http://www.genecodes.com). All of the genes encoding proteins, transfer RNAs (tRNAs), and ribosomal RNAs (rRNAs) were annotated on *Machilus* plastomes using the Dual Organellar Genome Annotator (DOGMA) software (Wyman et al., 2004). To further verify the identified tRNA genes, the tRNAscan-SE 1.21 program was used to predict their corresponding structures (Schattner et al., 2005). The genome map of *M. yunnanensis* and *M. balansae* was drawn by GenomeVx (Conant and Wolfe, 2008).

### Sliding Window Analysis of the Plastomes

After alignment using Clustal X 1.83 (Aiyar, 2000), the sequences were manually adjusted with Bioedit software (http://www.mbio.ncsu.edu/bioedit/bioedit.html). Further, we conducted a sliding window analysis to evaluate the variability (Pi) all over the plastomes in DnaSP version 5 software (Librado and Rozas, 2009). The window length was set to 600 base pairs and the step size was set as 200 base pairs.

### Mutation Events Analysis

To identify the microstructural mutations between *M. yunnanensis* and *M. balansae*, the two aligned sequences were further analyzed using DnaSP version 5 (Librado and Rozas, 2009) and MEGA version 5 (Tamura et al., 2011). Indel and SNP events were counted and positioned in the plastome using an R program. For the SSRs search, the minimum repeat unit was limited to eight for mononucleotides and four for dinucleotides. For non-SSR indel and SNP detection, the plastome of *M. yunnanensis* was used as a reference to determine the insertion or deletion events and transition (Ts) or transversion (Tv) events. In addition, the SNPs in the exon of the plastome were further classified into synonymous (S) and non-synonymous (N) substitutions. The gene classification was according to Chang et al. (2006).

## Results

### Size, Gene Content, and Organization of *M. yunnanensis* and *M. balansae* Plastomes

**FIGURE 1:**
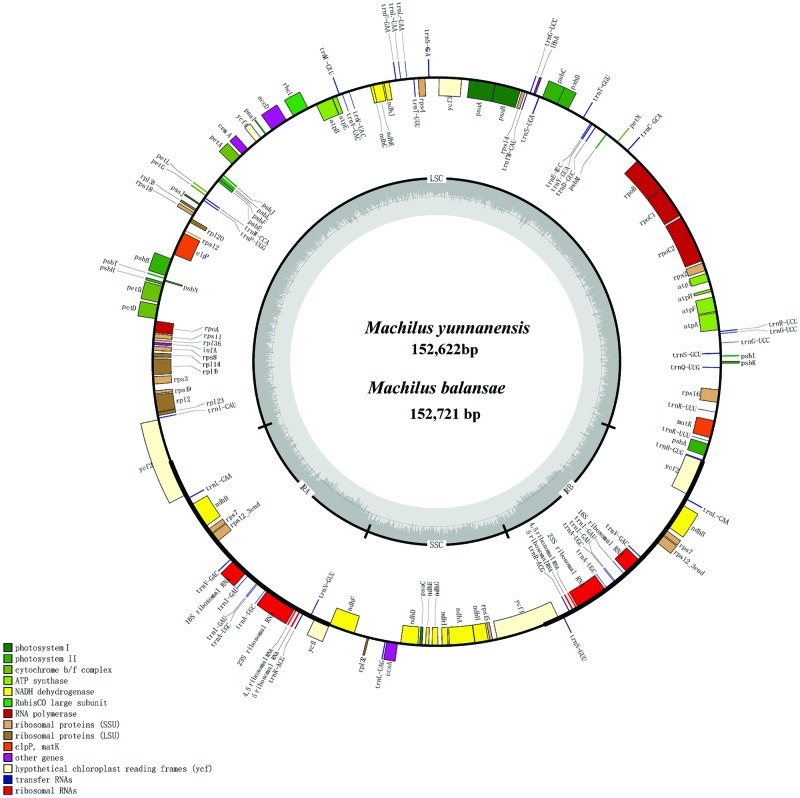
**Gene map of *Machilus yunnanensis* and *M. balansae* plastomes.** The annotation of the genome was performed using DOGMA. The genes that are drawn outside of the circle are transcribed clockwise, while those inside are counterclockwise. Small single copy (SSC), large single copy (LSC), and inverted repeats (IRa, IRb) are indicated.

The chloroplast genome of *M. yunnanensis* (deposited in GenBank: KT348516), with a length of 152, 622 bp, was 99 bp smaller than that of *M. balansae* (deposited in GenBank: KT348517) (**Figure 1**), and 78 bp smaller than that of *Cinnamomum kanehirae* (152,700 bp, GenBank accession No. KR014245) (Wu et al., 2015). All three are smaller than the genome of *Calycanthus fertilis* (153, 337 bp, GenBank accession No. NC_004993) in the Calycanthaceae, which is in the same order as the Lauraceae (Goremykin et al., 2003). A+T content is 61% in all four species. The *Machilus* chloroplast genomes include a pair of inverted repeats (IRs) of 20, 074 bp in *M. yunnanensis* and *M. balansae*, separated by a large single copy (LSC) region of 93, 675 bp in *M. yunnanensis* and 93, 676 bp in *M. balansae* and a small single copy (SSC) region of 18, 799 bp in *M. yunnanensis* and 18, 897 bp in *M. balansae* (**Table 1**). Both contain 113 different functional genes, including 79 protein-coding genes, 30 tRNA genes, and 4 rRNA genes (Supplementary Table S1). The gene map is shown in **Figure 1**. Among the functional genes, twelve protein-coding genes and six tRNA genes contain introns in both species (Supplementary Table S1).

**Table 1:** Summary of two complete plastomes of *Machilus*.

	*Machilus yunnanensis*	*Machilus balansae*
Total cpDNA size	152,622	152,721
Length of large single copy (LSC) region	93,675	93,676
Length of inverted repeat (IR) region	20,074	20,074
Length of small single copy (SSC) region	18,799	18,897
Total GC content (%)	39.16	39.15
LSC	37.93	37.95
IR	44.44	44.44
SSC	34.04	33.90
Total number of genes	113	113
Protein encoding	79	79
tRNA	30	30
rRNA	4	4

### Divergence Hotspots of *M. yunnanensis, M. balansae*, and *Cinnamomum kanehirae* Plastomes

**FIGURE 2:**
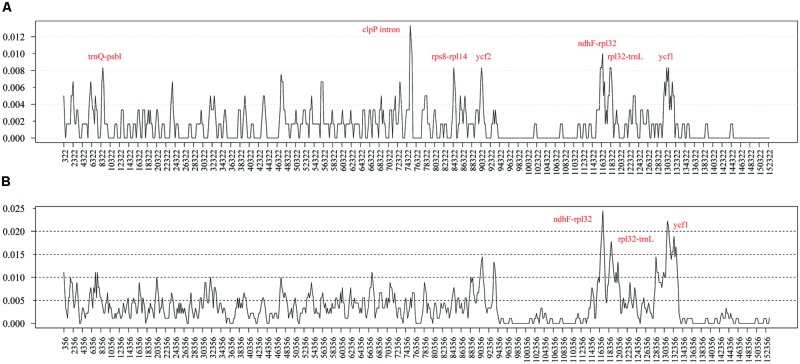
**Sliding window analysis of the whole plastomes of *M. yunnanensis* and *M. balansae***(A)** and those of *Cinnamomum kanehirae* and the two *Machilus* species **(B)**.** (window length: 600 bp, step size: 200 bp). X-axis: position of the midpoint of a window, Y-axis: nucleotide diversity of each window.

To elucidate the level of sequence divergence, the nucleotide variability (Pi) values within 600 bp in both chloroplast genomes of *M. yunnanensis, M. balansae*, and *Cinnamomum kanehirae* were calculated with DnaSP 5.0 software. Between two *Machilus* species these values varied from 0 to 0.01333 with a mean of 0.00154, indicating that the differences between the genomes were small. However, seven highly variable loci including the second intron of *clpP, ndhF-rpl32, trnQ-psbI, rps8-rpl14, ycf2, rpl32-trnL*, and *ycf1* were precisely located (**Figure 2A**). All of these regions had much higher values than other regions (Pi > 0.008). Three of these loci lie in the LSC region, three in the SSC region, and one in the IR region. Among them, the introns of *clpP, rpl32-trnL*, and *ycf1* loci have been reported before as highly variable regions in seed plants (Dong et al., 2012), while *ndhF-rpl32, trnQ-psbI, rps8-rpl14*, and *ycf2* loci seem to be especially variable in *Machilus*. Among *Cinnamomum kanehirae* and the two *Machilus* species the Pi values varied from 0 to 0.02444, indicating that the differences between the two genera were larger than those between congeneric species. Three of the seven loci, including *ndhF-rpl32, rpl32-trnL*, and *ycf1*, were particularly highly variable between *Cinnamomum* and *Machilus* species (Pi > 0.015; **Figure 2B**).

### Number and Forms of Microstructural Mutations

Indel markers display no ambiguity in complex mutation patterns with the advantages of low cost and high precision. To detect more variable sites in *Machilus*, indel mutations between the chloroplast genomes of *M. yunnanensis* and *M. balansae* were compared. There are 52 indels in gene spacer regions, 12 indels in introns, and one in the exon of *ndhF*. Further, these indels were classified into 36 simple sequences repeat (SSR) indels (**Table 2**) and 29 non-SSR indels (**Table 3**). For the SSR indels, there are 34 single nucleotide repeats A/T ranged from 8 to 19 bp, one 9 bp single nucleotide repeat C in the *petA-psbJ* gene spacer region, and one double nucleotide repeat AT with 5 and 7 bp in the *petN-psbM* region (**Table 2**). The sizes of most non-SSR indels ranged from 1 to 7 bp, while that within the *ndhF-rpl32, trnH-psbA*, and *psbM-trnD* gene spacer sequences were 94 bp, 22 bp, and 21 bp, respectively (**Table 3**). Of all of these indel events, 83.08% of sites were in the LSC region while 3.07% of sites were in the IR regions. In addition, one micro-inversion event with five bases was detected in the *ccsA-ndhD* gene spacer region (Supplementary Figure S3).

**Table 2:** Location of simple sequence repeats in the *Machilus* plastomes.

No.	Location	Region	Motif	No. of Repeats	
				*M. yunnanensis*	*M. balansae*
1	*trnK-rps16*	Intergenic	T	12	14
2	*trnK-rps16*	Intergenic	T	9	10
3	*rps16*	Intron	T	9	10
4	*rps16*	Intron	A	14	10
5	*rps16-trnQ*	Intergenic	T	11	18
6	*trnS-trnG*	Intergenic	T	8	9
7	*trnG-trnG*	Intergenic	T	10	11
8	*rps2-rpoC2*	Intergenic	T	12	10
9	*petN-psbM*	Intergenic	AT	5	7
10	*trnD-trnY*	Intergenic	A	10	11
11	*trnT-psbD*	Intergenic	T	8	7
12	*trnG-trnfM*	Intergenic	A	18	19
13	*psaA-ycf3*	Intergenic	T	13	15
14	*ycf3*	Intron	T	11	10
15	*trnT-trnL*	Intergenic	A	12	11
16	*ndhC-trnV*	Intergenic	T	11	12
17	*trnM-atpE*	Intergenic	T	14	10
18	*psaI-ycf4*	Intergenic	A	7	6
19	*petA-psbJ*	Intergenic	C	8	9
20	*psbE-petL*	Intergenic	T	8	9
21	*psbE-petL*	Intergenic	T	9	8
22	*rps18-rpl20*	Intergenic	T	12	15
23	*rpl20-rps12*	intergenic	A	14	13
24	*clpP*	Intron	T	11	9
25	*clpP*	Intron	T	12	13
26	*clpP*	Intron	A	17	12
27	*clpP*	Intron	T	14	13
28	*petB-petB*	Intergenic	A	9	10
29	*rps8-rpl14*	Intergenic	T	13	11
30	*rpl14-rpl16*	Intergenic	T	15	16
31	*rpl16-rps3*	Intergenic	T	10	12
32	*rps3-rps19*	Intergenic	T	9	8
33	*rps19-rpl2*	Intergenic	T	9	10
34	*rpl32-trnL*	Intergenic	T	11	15
35	*ccsA-ndhD*	Intergenic	T	10	12
36	*rps15-ycf1*	Intergenic	T	14	13

**Table 3:** Forms and numbers of indel mutation events in the plastome between the two *Machilus* species.

No.	Location	region	Motif	Size	Driection^a^
1	*trnH-psbA*	Intergenic	aaaacaaaatgttgtacataaa	22	Deletion
2	*rps16-trnQ*	Intergenic	cttgta	6	Deletion
3	*trnG*	Intron	c	1	Insertion
4	*trnG*	Intron	tga	3	Deletion
5	*atpF*	Intron	tg	2	Deletion
6	*psbM-trnD*	Intergenic	tacatggaccaggagcaatcg	21	Insertion
7	*trnE-trnT*	Intergenic	taatt	5	Insertion
8	*ycf3-trnS*	Intergenic	tgtat	5	Deletion
9	*trnS-rps4*	Intergenic	g	1	Deletion
10	*trnS-rps4*	Intergenic	aagag	5	Insertion
11	*ndhC-trnV*	Intergenic	attaaat	7	Deletion
12	*ndhC-trnV*	Intergenic	a	1	Deletion
13	*trnV*	Intron	t	1	Deletion
14	*ycf4-cemA*	Intergenic	ttctat	6	Insertion
15	*rpl16*	Intron	ggat	4	Deletion
16	*rpl2-rpl2*	Intergenic	tc	2	Insertion
17	*ycf1-ndhF*	Intergenic	a	1	Deletion
18	*ndhF*	Exon	ttcgaa	6	Insertion
19	*ndhF-rpl32*	Intergenic	aatcaagatatacaagatataaaagaact caaatatgatttttcattcttaattattctgatt ctttccaaactattgaaaaaaaaaaaaaaac	94	Deletion
20	*ndhF-rpl32*	Intergenic	t	1	Deletion
21	*rpl32-trnL*	Intergenic	g	1	Deletion
22	*rps15-ycf1*	Intergenic	a	1	Deletion
23	*rpl23-rpl2*	Intergenic	ag	2	Insertion
24	*atpF-atpH*	Intergenic	c	1	Insertion
25	*ycf3-trnS*	Intergenic	a	1	Insertion
26	*ndhC-trnV*	Intergenic	a	1	Insertion
27	*rpl32-trnL*	Intergenic	t	1	Deletion
28	*rbcL-accD*	Intergenic	t	1	Insertion
29	*rbcL-accD*	Intergenic	t	1	Insertion

*^a^The plastome of *M. yunnanensis* was used as a reference.*

### Numbers and Pattern of SNP Mutations

**FIGURE 3:**
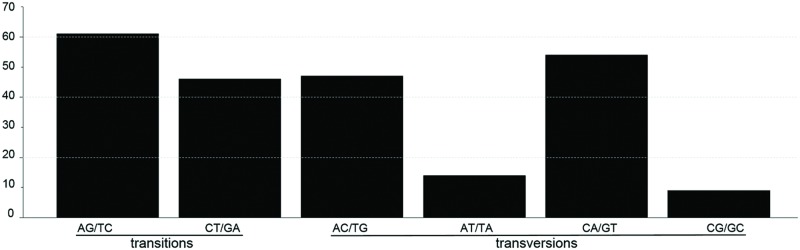
**The patterns of nucleotide substitutions among *M. yunnanensis* and *M. balansae* plastomes.** The patterns were divided into six types as indicated by the six non-strand-specific base-substitution types (i.e., numbers of considered G to A and C to T sites for each respective set of associated mutation types). The plastome of *M. yunnanensis* was used as a reference.

SNP markers are the most abundant type of mutations, but have never been screened in *Machilus* species. We detected 95 SNPs, including 48 Ts and 47 Tv, in gene coding regions (**Figure 3**) and 136 SNPs, including 58 Ts and 78 Tv, in non-coding regions (Supplementary Table S2). The Tv to Ts ratio was 1: 0.74. Among the Tv, 14 were Tv between T and A, 9 were Tv between C and G, and the other 101 were related to GC content changes. Among substitution events in the gene coding regions, non-synonymous and synonymous substitutions shared 48 and 47 of 95 sites in the entire plastomes, and 23 of 79 genes had non-synonymous substitution sites. However, two genes *psaA* and *ycf1* had more non-synonymous than synonymous substitutions sites, suggesting these two genes had a relatively high evolution rate (**Table 4**). In all of these substitution events, 74.03% of SNP sites were in the LSC region while 1.73% of SNP sites were in the IR regions.

**Table 4:** Comparisons of mutational changes, number of transitions (Ts) and transversions (Tv), synonymous (S), and non-synonymous (N) substitutions per gene of protein coding chloroplast genes between *M. yunnanensis* and *M. balansae*.

	Gene	Ts	Tv	S	N
Photosynthetic apparatus	*psaA*	2	2	1	3
	*psaB*	0	1	1	0
	*psaJ*	2	0	2	0
	*psbA*	1	0	0	1
	*psbL*	1	0	0	1
	*psbT*	1	1	2	0
	*petA*	1	0	1	0
	*ycf4*	0	1	0	1
	
	**Total**	**8**	**5**	**7**	**6**
Photosynthetic metabolism	*atpA*	1	0	1	0
	*atpB*	1	1	1	1
	*atpE*	3	1	4	0
	*atpF*	0	1	0	1
	*atpI*	1	0	1	0
	*ndhA*	0	2	0	2
	*ndhD*	1	0	0	1
	*ndhE*	1	1	1	1
	*ndhF*	2	1	3	0
	*ndhG*	3	0	1	2
	*ndhH*	1	0	1	0
	*ndhK*	2	1	1	2
	*rbcL*	0	2	1	1
	
	**Total**	**16**	**10**	**15**	**11**
Gene expression	*rpl2*	1	0	1	0
	*rpl14*	2	2	2	2
	*rpoA*	0	1	0	1
	*rpoB*	1	1	0	2
	*rpoC1*	1	0	1	0
	*rpoC2*	4	3	5	2
	*rps3*	1	1	1	1
	*rps4*	1	0	1	0
	*rps14*	1	0	1	0
	*rps18*	0	1	0	1
	
	**Total**	**12**	**9**	**12**	**9**
Other Genes	*ycf1*	6	12	6	12
	*ycf2*	3	6	4	5
	*ccsA*	0	2	1	1
	*cemA*	1	1	1	1
	*matK*	2	2	2	2
	
	**Total**	**48**	**47**	**48**	**47**

## Discussion

This study produced two complete chloroplast genomes for species in the Lauraceae, which comprises nearly 3500 species in over 50 genera worldwide. For species identification and population structure analysis in this family, the rapidly developed molecular markers such as indels and SNPs have been proved to have significant potential. Global alignment of 13 *Gossypium* plastomes (Malvaceae) indicated that the total number of SNPs varied from 6 to 1000 and the number of indels ranged from 3 to 178, which supported that the plastome divergence was approximate 0.00159 to 0.00454 within allotetraploids of *Gossypium* (Xu et al., 2012). Plastome comparative analysis of five *Camellia* species identified 15 molecular markers with over 1.5% sequence divergences, which were used to promote the further phylogenetic analysis and species identification of *Camellia* species (Huang et al., 2014). The indel and SNP variable sites of plastomes of 12 Triticeae species were used to estimate that barley diverged from rye and wheat around 8.5 million years ago and rye diverged from *Triticum aestivum* around 3.5 million years ago (Middleton et al., 2014). These results show that molecular markers including indels and SNPs are useful tools in research.

Out of the 65 indel markers between *M. yunnanensis* and *M. balansae* plastomes, the largest indel is located within the intergenic sequence *ndhF-rpl32* (94 bp) in the SSC region. Another two large indels were found within *trnH-psbA* (22 bp) and *psbM-trnD* (21 bp) in the LSC region. Previous work in other plants has identified large indels in intergenic spacers, such as *ndhF*–*rpl32, rpoB*–*trnC, trnE-trnT, rpl32-trnL, trnQ-rps16*, and protein coding genes, such as *accD, rpl20, ycf1*, and *ycf15* (Shaw et al., 2007; Nashima et al., 2015). Most of these large indel events occurred in single copy regions but not IR regions. The 36 SSR loci identified in this study may be useful in population and evolutionary studies as well, as they were in *Panax ginseng* (Kim and Lee, 2004a), *Cucumis sativus* (Kim et al., 2006), *Vigna radiata* (Tangphatsornruang et al., 2010), and *Pyrus pyrifolia* (Terakami et al., 2012). In addition, one micro-inversion of five nucleotides was detected between *M. yunnanensis* and *M. balansae* plastomes, which indicate that differences in micro-inversion events could exist between *Machilus* species as previously report in *Solanum* species (Kim and Lee, 2004b; Gargano et al., 2012).

Besides the indel markers, 231 SNP markers were detected between *M. yunnanensis* and *M. balansae* plastomes, which indicated that the nucleotide substitution events in the chloroplast genome of *Machilus* species are more than that between species of rice and less than species of ginseng, potato, and orange. Comparative analysis of genomes found 159 SNP sites between two chloroplast genomes of *O. sativa* and *O. nivara* (Masood et al., 2004), 464 between plastomes of *P. ginseng* and *P. notoginseng* (Dong et al., 2014), 591 between plastomes of *Solanum tuberosum* and *S. bulbocastanum* (Chung et al., 2006), and 330 between plastomes of *Citrus sinensis* and *C. aurantiifolia* (Su et al., 2014). For the 95 SNP markers in gene coding regions, non-synonymous and synonymous substitutions shared similar numbers of 48 and 47 sites in the entire *Machilus* plastomes, implying that constraint mechanisms of substitution existed. Under the constraint background, photosynthetic metabolism genes *atpE* and *ndhF* and gene expression genes *rpoC2* and *rpl2* shared the extra synonymous substitution sites which are equal with the non-synonymous substitution sites of *ycf1, ycf2*, and photosynthetic apparatus gene *psaA*.

The Indel and SNP mutation events in the genome were not random but clustered as “hotspots” (Shaw et al., 2007; Worberg et al., 2007). Such mutational dynamics created the highly variable regions in the genome. In *M. yunnanensis* and *M. balansae* plastomes, we identified seven highly variable loci including the second intron of *clpP, ndhF-rpl32, trnQ-psbI, rps8-rpl14, ycf2, rpl32-trnL*, and *ycf1*. Three of the seven, including *ndhF-rpl32, rpl32-trnL*, and *ycf1*, were particularly highly variable between *Machilus* and *Cinnamomum* plastomes. The second intron of *clpP, rpl32-trnL*, and *ycf1* were the focus of previous analyses investigating sequence variation in seed plants (Dong et al., 2012, 2015; Sarkinen and George, 2013). The *ycf2* and *ndhF-rpl32* loci have also been widely used for phylogenetic studies (Shaw et al., 2007; Huang et al., 2010). Here, two rarely reported highly variable loci *rps8-rpl14* and *trnQ-psbI* were present in *Machilus* plastomes. In contrast, none of the 14 regions in the chloroplast genome used previously for phylogenetic analysis were found to be variable (Rohwer et al., 2009; Li et al., 2011). All of these seven highly variable regions and the indel or SNP markers are better to use for phylogenetic studies at the species level in *Machilus*. We encourage researchers working on the Lauraceae family to use the seven highly variable regions identified in this study for phylogenetic analysis.

## Conflict of Interest Statement

The authors declare that the research was conducted in the absence of any commercial or financial relationships that could be construed as a potential conflict of interest.

## References

[B1] Aiyar A. (2000). The use of CLUSTAL W and CLUSTAL X for multiple sequence alignment. *Methods Mol. Biol.* 132 221–241. 10.1385/1-59259-192-2:221 10547838

[B2] Chang C. C. Lin H. C. Lin I. P. Chow T. Y. Chen H. H. Chen W. H. (2006). The chloroplast genome of *Phalaenopsis aphrodite* (Orchidaceae): comparative analysis of evolutionary rate with that of grasses and its phylogenetic implications. *Mol. Biol. Evol.* 23 279–291. 10.1093/molbev/msj029 16207935

[B3] Chen J. Q. Li L. Li J. Li X. W. (2009). Bayesian inference of nrDNA ITS sequences from *Machilus* (Lauraceae) and its systematic significance. *Acta Bot. Yunnan.* 31 117–126. 10.3724/SP.J.1143.2009.08206

[B4] Chung H. J. Jung J. D. Park H. W. Kim J. H. Cha H. W. Min S. R. (2006). The complete chloroplast genome sequences of *Solanum tuberosum* and comparative analysis with *Solanaceae* species identified the presence of a 241-bp deletion in cultivated potato chloroplast DNA sequence. *Plant Cell Rep.* 25 1369–1379. 10.1007/s00299-006-0196-4 16835751

[B5] Conant G. C. Wolfe K. H. (2008). GenomeVx: simple web-based creation of editable circular chromosome maps. *Bioinformatics* 24 861–862. 10.1093/bioinformatics/btm598 18227121

[B6] Dong W. Liu H. Xu C. Zuo Y. Chen Z. Zhou S. (2014). A chloroplast genomic strategy for designing taxon specific DNA mini-barcodes: a case study on ginsengs. *BMC Genet.* 15:138 10.1186/s12863-014-0138-z PMC4293818 25526752

[B7] Dong W. Liu J. Yu J. Wang L. Zhou S. (2012). Highly variable chloroplast markers for evaluating plant phylogeny at low taxonomic levels and for DNA barcoding. *PLoS ONE* 7:e35071 10.1371/journal.pone.0035071 PMC3325284 22511980

[B8] Dong W. Xu C. Cheng T. Lin K. Zhou S. (2013). Sequencing angiosperm plastid genomes made easy: a complete set of universal primers and a case study on the phylogeny of saxifragales. *Genome Biol. Evol.* 5 989–997. 10.1093/gbe/evt063 23595020 PMC3673619

[B9] Dong W. Xu C. Li C. Sun J. Zuo Y. Shi S. (2015). ycf1, the most promising plastid DNA barcode of land plants. *Sci. Rep.* 5 8348 10.1038/srep08348 PMC4325322 25672218

[B10] Doyle J. J. Davis J. I. Soreng R. J. Garvin D. Anderson M. J. (1992). Chloroplast DNA inversions and the origin of the grass family (Poaceae). *Proc. Natl. Acad. Sci. U.S.A.* 89 7722–7726. 10.1073/pnas.89.16.7722 1502190 PMC49783

[B11] Gargano D. Scotti N. Vezzi A. Bilardi A. Valle G. Grillo S. (2012). Genome-wide analysis of plastome sequence variation and development of plastidial CAPS markers in common potato and related *Solanum* species. *Genet. Resour. Crop Evol.* 59 419–430. 10.1007/s10722-011-9692-7

[B12] Goremykin V. Hirsch-Ernst K. I. Wolfl S. Hellwig F. H. (2003). The chloroplast genome of the basal angiosperm *Calycanthus fertilis* - structural and phylogenetic analyses. *Plant Syst. Evol.* 242 119–135. 10.1007/s00606-003-0056-4

[B13] Huang H. Shi C. Liu Y. Mao S. Y. Gao L. Z. (2014). Thirteen *Camellia chloroplast* genome sequences determined by high-throughput sequencing: genome structure and phylogenetic relationships. *BMC Evol. Biol.* 14:151 10.1186/1471-2148-14-151 PMC4105164 25001059

[B14] Huang J. L. Sun G. L. Zhang D. M. (2010). Molecular evolution and phylogeny of the angiosperm ycf2 gene. *J. Syst. Evol.* 48 240–248. 10.1111/j.1759-6831.2010.00080.x

[B15] Ingvarsson P. K. Ribstein S. Taylor D. R. (2003). Molecular evolution of insertions and deletion in the chloroplast genome of Silene. *Mol. Biol. Evol.* 20 1737–1740. 10.1093/molbev/msg163 12832644

[B16] Jansen R. K. Palmer J. D. (1987). A chloroplast DNA inversion marks an ancient evolutionary split in the sunflower family (Asteraceae). *Proc. Natl. Acad. Sci. U.S.A.* 84 5818–5822. 10.1073/pnas.84.16.5818 16593871 PMC298954

[B17] Kim J. S. Jung J. D. Lee J. A. Park H. W. Oh K. H. Jeong W. J. (2006). Complete sequence and organization of the cucumber (*Cucumis sativus* L. cv. Baekmibaekdadagi) chloroplast genome. *Plant Cell Rep.* 25 334–340. 10.1007/s00299-005-0097-y 16362300

[B18] Kim K. J. Lee H. L. (2004a). Complete chloroplast genome sequences from Korean ginseng (Panax schinseng Nees) and comparative analysis of sequence evolution among 17 vascular plants. *DNA Res.* 11 247–261. 10.1093/dnares/11.4.247 15500250

[B19] Kim K. J. Lee H. L. (2004b). Widespread occurrence of small inversions un the chloroplast genomes of land plants. *Mol. Cells* 19 104–113. 15750347

[B20] Li J. Wang S. Jing Y. Wang L. Zhou S. (2013). A modified CTAB protocol for plant DNA extraction. *Chin. Bull. Bot.* 48 72–78. 10.3724/SP.J.1259.2013.00072

[B21] Li L. Li J. Rohwer J. G. van der Werff H. Wang Z. H. Li H. W. (2011). Molecular phylogenetic analysis of the Persea group (Lauraceae) and its biogeographic implications on the evolution of tropical and subtropical Amphi-Pacific Disjunctions. *Am. J. Bot.* 98 1520–1536. 10.3732/ajb.1100006 21860056

[B22] Librado P. Rozas J. (2009). DnaSP v5: a software for comprehensive analysis of DNA polymorphism data. *Bioinformatics* 25 1451–1452. 10.1093/bioinformatics/btp187 19346325

[B23] Masood M. S. Nishikawa T. Fukuoka S. Njenga P. K. Tsudzuki T. Kadowaki K. (2004). The complete nucleotide sequence of wild rice (*Oryza nivara*) chloroplast genome: first genome wide comparative sequence analysis of wild and cultivated rice. *Gene* 340 133–139. 10.1016/j.gene.2004.06.008 15556301

[B24] Middleton C. P. Senerchia N. Stein N. Akhunov E. D. Keller B. Wicker T. (2014). Sequencing of chloroplast genomes from wheat, barley, rye and their relatives provides a detailed insight into the evolution of the Triticeae tribe. *PLoS ONE* 9:e85761 10.1371/journal.pone.0085761 PMC3948623 24614886

[B25] Nashima K. Terakami S. Nishitani C. Kunihisa M. Shoda M. Takeuchi M. (2015). Complete chloroplast genome sequence of pineapple (*Ananas comosus*). *Tree Genet. Genomes* 11 60 10.1007/s11295-015-0892-8

[B26] Rohwer J. G. (2000). Toward a phylogenetic classification of the lauraceae: evidence from matK sequences. *Syst. Bot.* 25 60–71. 10.2307/2666673

[B27] Rohwer J. G. Li J. Rudolph B. Schmidt S. A. van der Werff H. Li H. W. (2009). Is Persea (Lauraceae) monophyletic? Evidence from nuclear ribosomal ITS sequences. *Taxon* 58 1153–1167.

[B28] Rohwer J. G. Rudolph B. (2005). Jumping genera: the phylogenetic positions of Cassytha, Hypodaphnis, and Neocinnamomum (Lauraceae) based on different analyses of trnK intron sequences. *Ann. Mo. Bot. Gard.* 92 153–178.

[B29] Sarkinen T. George M. (2013). Predicting plastid marker variation: can complete plastid genomes from closely related species help? *PLoS ONE* 8:e82266 10.1371/journal.pone.0082266 PMC3843732 24312409

[B30] Schattner P. Brooks A. N. Lowe T. M. (2005). The tRNAscan-SE, snoscan and snoGPS web servers for the detection of tRNAs and snoRNAs. *Nucleic Acids Res.* 33 W686–W689. 10.1093/nar/gki366 15980563 PMC1160127

[B31] Shaw J. Lickey E. B. Schilling E. E. Small R. L. (2007). Comparison of whole chloroplast genome sequences to choose noncoding regions for phylogenetic studies in angiosperms: the tortoise and the hare III. *Am. J. Bot.* 94 275–288. 10.3732/ajb.94.3.275 21636401

[B32] Su H. J. Hogenhout S. A. Al-Sadi A. M. Kuo C. H. (2014). Complete chloroplast genome sequence of omani lime (*Citrus aurantiifolia*) and comparative analysis within the Rosids. *PLoS ONE* 9:e113049 10.1371/journal.pone.0113049 PMC4232571 25398081

[B33] Tamura K. Peterson D. Peterson N. Stecher G. Nei M. Kumar S. (2011). MEGA5: molecular evolutionary genetics analysis using maximum likelihood, evolutionary distance, and maximum parsimony methods. *Mol. Biol. Evol.* 28 2731–2739. 10.1093/molbev/msr121 21546353 PMC3203626

[B34] Tang J. B. Xia H. A. Cao M. L. Zhang X. Q. Zeng W. Y. Hu S. N. (2004). A comparison of rice chloroplast genomes. *Plant Physiol.* 135 412–420. 10.1104/pp.103.031245 15122023 PMC429394

[B35] Tang S. C. Xu W. B. Wei F. N. (2010). Machilus parapauhoi sp. nov. and a new synonym of *Machilus* (Lauraceae) from east Asia. *Nord. J. Bot.* 28 503–505. 10.1111/j.1756-1051.2010.00748.x

[B36] Tangphatsornruang S. Sangsrakru D. Chanprasert J. Uthaipaisanwong P. Yoocha T. Jomchai N. (2010). The chloroplast genome sequence of mungbean (*Vigna radiata*) determined by high-throughput pyrosequencing: structural organization and phylogenetic relationships. *DNA Res.* 17 11–22. 10.1093/dnares/dsp025 20007682 PMC2818187

[B37] Terakami S. Matsumura Y. Kurita K. Kanamori H. Katayose Y. Yamamoto T. (2012). Complete sequence of the chloroplast genome from pear (*Pyrus pyrifolia*): genome structure and comparative analysis. *Tree Genet. Genomes* 8 1–14. 10.1007/s11295-012-0469-8

[B38] Wei F. N. Werff H. V. D. (2008). “Flora of China,” in *Dipterocarpaceae* Vol. 13 eds Wu Z. Y. Raven P. H. Hong D. Y. (St. Louis, MO: Science Press, Beijing, and Missouri Botanical Garden Press) 201–224.

[B39] Worberg A. Quandt D. Barniske A. M. Lohne C. Hilu K. W. Borsch T. (2007). Phylogeny of basal eudicots: insights from non-coding and rapidly evolving DNA. *Organ. Diver Evol.* 7 55–77. 10.1016/j.ode.2006.08.001

[B40] Wu C. C. Ho C. K. Chang S. H. (2015). The complete chloroplast genome of Cinnamomum kanehirae Hayata (Lauraceae). *Mitochondr. DNA* 8 1–2. 10.3109/19401736.2015.1043541 26053940

[B41] Wyman S. K. Jansen R. K. Boore J. L. (2004). Automatic annotation of organellar genomes with DOGMA. *Bioinformatics* 20 3252–3255. 10.1093/bioinformatics/bth352 15180927

[B42] Xu Q. Xiong G. Li P. He F. Huang Y. Wang K. (2012). Analysis of complete nucleotide sequences of 12 gossypium chloroplast genomes: origin and evolution of allotetraploids. *PLoS ONE* 7:e37128 10.1371/journal.pone.0037128 PMC3411646 22876273

[B43] Zhao Y. B. Yin J. L. Guo H. Y. Zhang Y. Y. Xiao W. Sun C. (2015). The complete chloroplast genome provides insight into the evolution and polymorphism of *Panax ginseng*. *Front. Plant Sci.* 5:696 10.3389/fpls.2014.00696 PMC4294130 25642231

