# Supplementary material for: Comparative analysis of complete chloroplast genome sequences of two tropical trees Machilus yunnanensis and Machilus balansae in the family Lauraceae
Source: Front Plant Sci. 2015 Aug 25;6:662. doi: 10.3389/fpls.2015.00662 (PMC4548089; doi:10.3389/fpls.2015.00662)
Supplement: Supplementary file 3 [file Image_1.PDF]

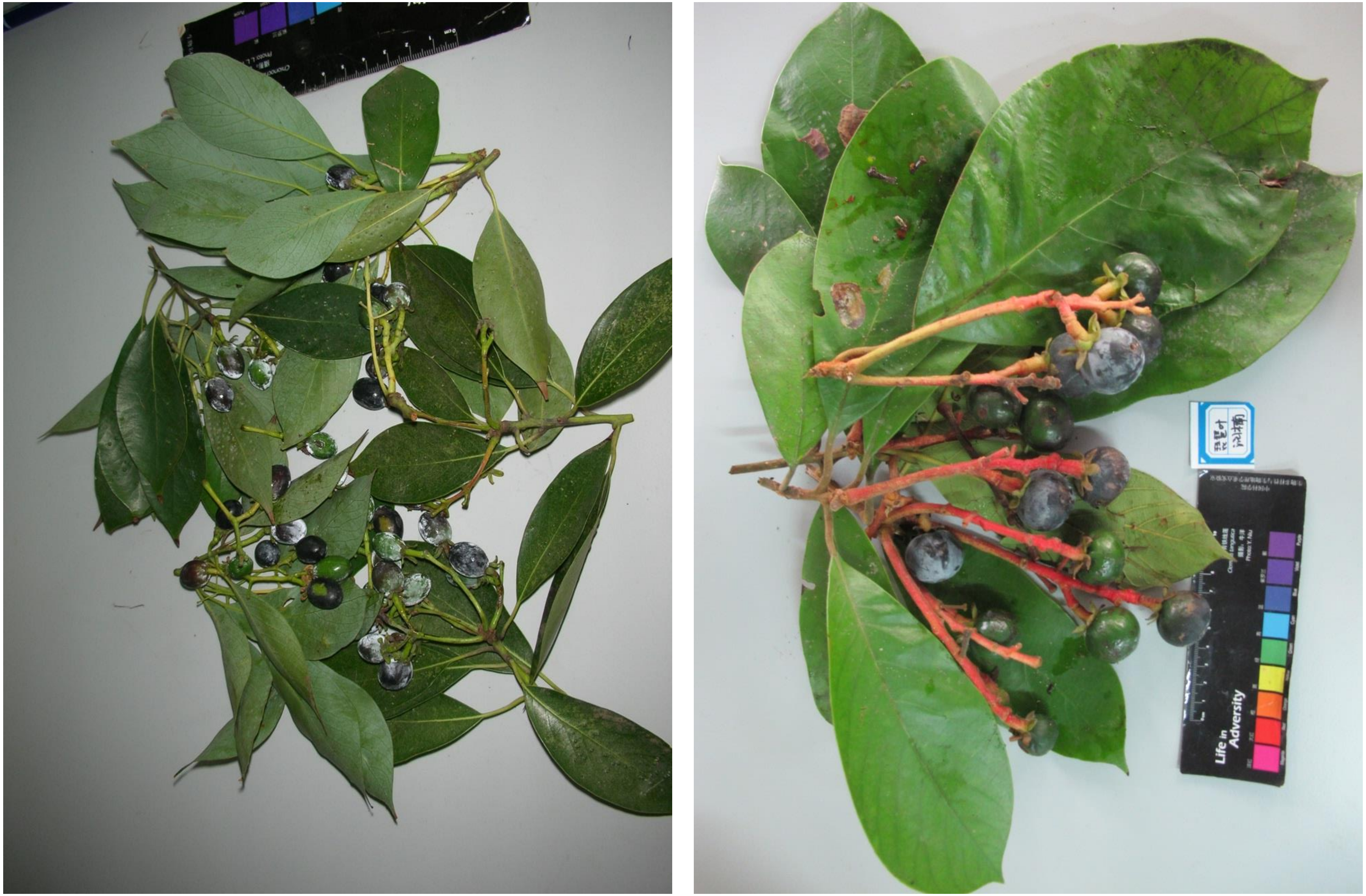

A. B.

Figure S1. The fruitful branches of *M. yunnanensis* (A) and *M. balansae* (B).

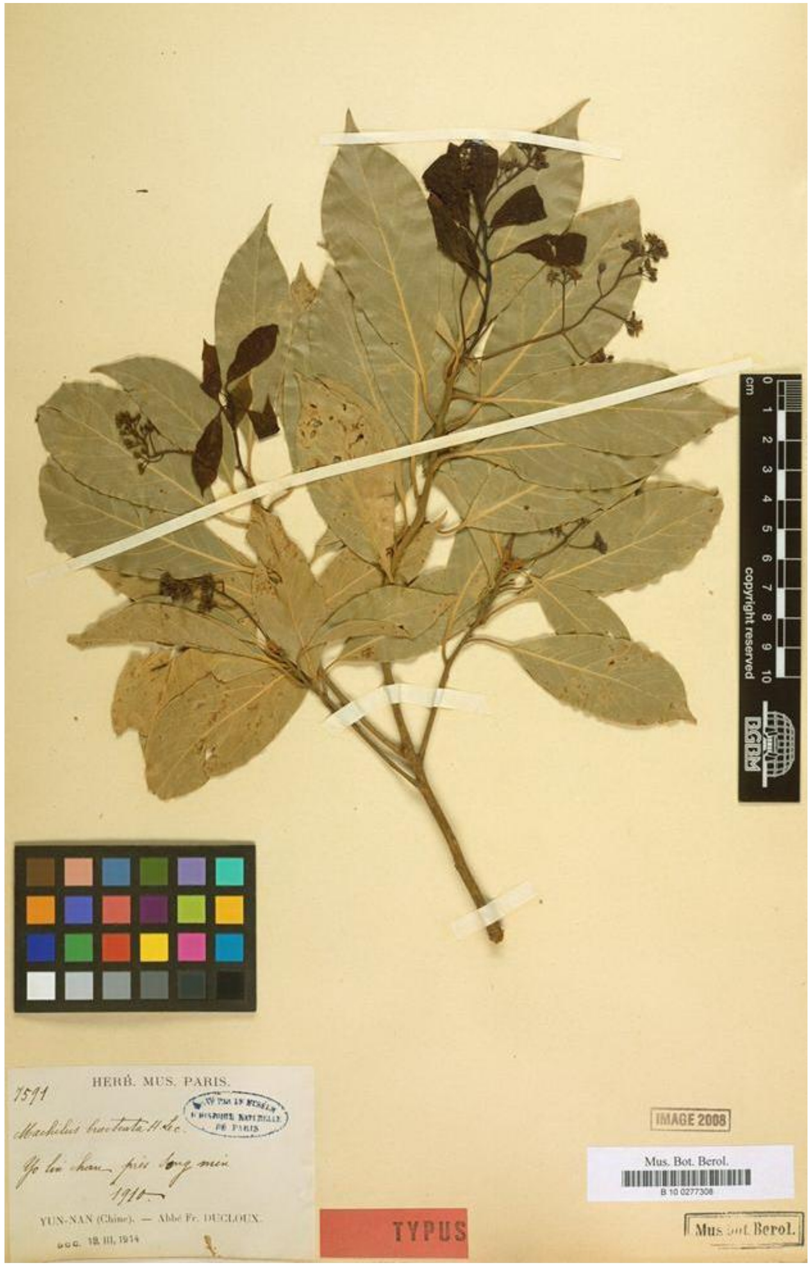

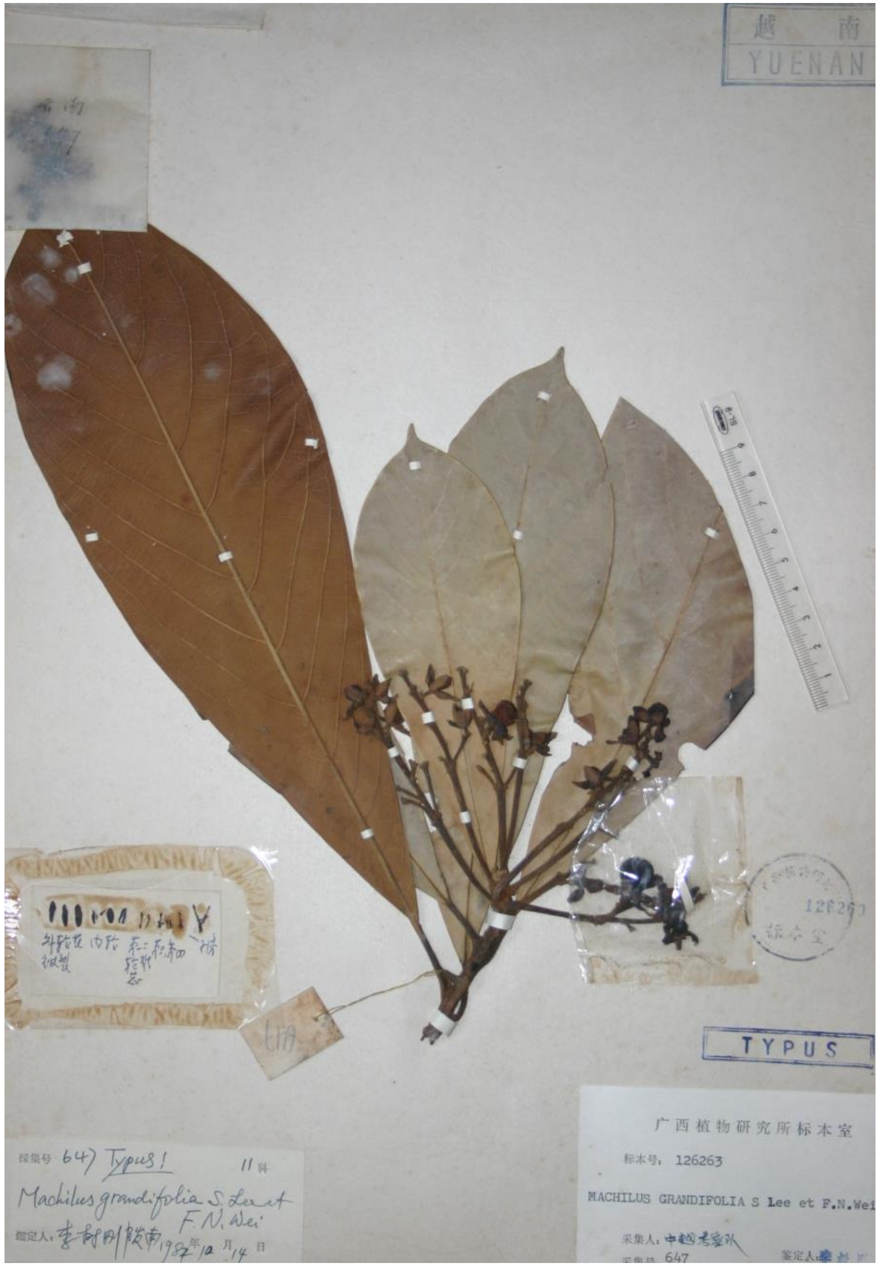

A. B.

Figure S2. The typus photos of *M. yunnanensis* (A) and *M. balansae* (B). *M. grandifolia* was regarded as a new synonym of *M. balansae* in Tang et al., 2010.

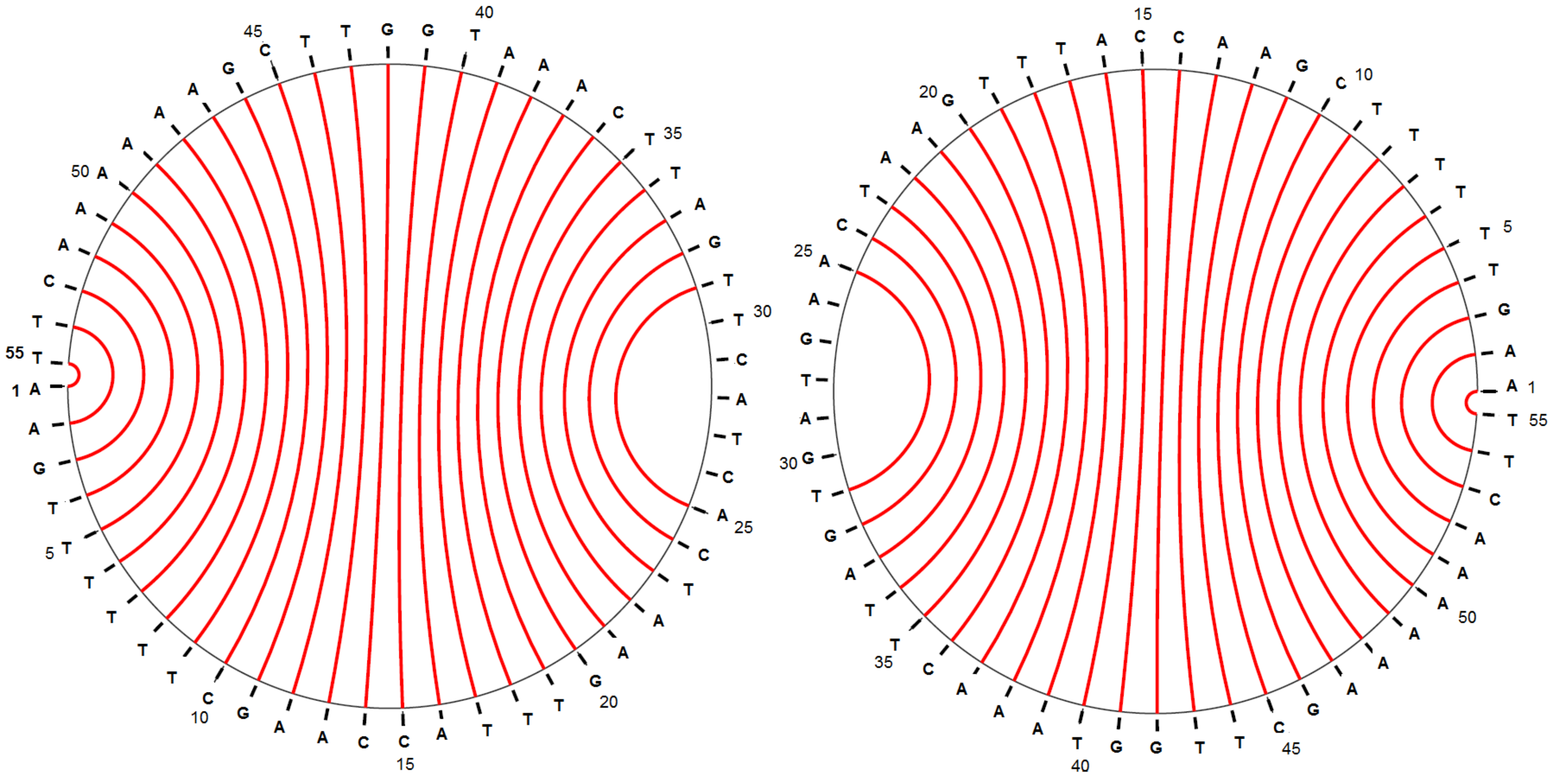

Figure S3. The hairpin loops of micro-inversion in the plastomes of *M. yunnanensis* (left) and *M. balansae* (right).
